# Synthesis and Comparative Studies of Glucose Oxidase Immobilized on Fe_3_O_4_ Magnetic Nanoparticles Using Different Coupling Agents

**DOI:** 10.3390/nano12142445

**Published:** 2022-07-17

**Authors:** Alina Gabriela Rusu, Aurica P. Chiriac, Loredana Elena Nita, Vera Balan, Alexandru Mihail Serban, Alexandra Croitoriu

**Affiliations:** 1 Natural Polymers, Bioactive and Biocompatible Materials Department, Petru Poni Institute of Macromolecular Chemistry, 41A Gr. Ghica–Voda Alley, 700487 Iasi, Romania; achiriac@icmpp.ro (A.P.C.); lnazare@icmpp.ro (L.E.N.); serban.alexandru@icmpp.ro (A.M.S.); croitoriu.alexandra@icmpp.ro (A.C.); 2 Faculty of Medical Bioengineering, Grigore T. Popa University of Medicine and Pharmacy of Iasi, 700115 Iasi, Romania; balanvera@yahoo.com

**Keywords:** magnetic nanoparticles, enzyme, immobilization

## Abstract

Squaric acid (SA) is a compound with potential to crosslink biomacromolecules. Although SA has become over the last years a well-known crosslinking agent as a result of its good biocompatibility, glutaraldehyde (GA), a compound with proven cytotoxicity is still one of the most used crosslinkers to develop nanomaterials. In this regard, the novelty of the present study consists in determining whether it may be possible to substitute GA with a new bifunctional and biocompatible compound, such as SA, in the process of enzyme immobilization on the surface of magnetic nanoparticles (MNPs). Thus, a direct comparison between SA- and GA-functionalized magnetic nanoparticles was realized in terms of physico-chemical properties and ability to immobilize catalytic enzymes. The optimal conditions of the synthesis of the two types of GOx-immobilized MNPs were described, thus emphasizing the difference between the two reagents. Scanning Electron Microscopy and Dynamic Light Scattering were used for size, shape and colloidal stability characterization of the pristine MNPs and of those coupled with GOx. Binding of GOx to MNPs by using GA or SA was confirmed by FT-IR spectroscopy. The stability of the immobilized and free enzyme was investigated by measuring the enzymatic activity. The study confirmed that the resulting activity of the immobilized enzyme and the optimization of enzyme immobilization depended on the type of reagent used and duration of the process. The catalytic performance of immobilized enzyme was tested, revealing that the long-term colloidal stability of SA-functionalized MNPs was superior to those prepared with GA. In conclusion, the SA-functionalized bioconjugates have a better potential as compared to the GA-modified nanosystems to be regarded as catalytic nanodevices for biomedical purposes such as biosensors.

## 1. Introduction

All living species contain enzymes which catalyze a multitude of different biochemical reactions taking place inside the biological cells [1]. Enzymes are defined as biologically active proteins [2], but often they are endowed with a non-protein component known as a cofactor which is responsible for catalytic activity. They are biocompatible and biodegradable, and due to their ease of production and substrate specificity, they are widely used in clinical diagnosis [3] and the environmental industry [4]. However, all these remarkable characteristics of enzymes and their widespread use in various fields of application are often hampered by their unstable structure, short lifetime, separation problems and high costs in enzymatic recycling [5,6]. Although many techniques such as modification or protein engineering have been employed to improve the enzyme catalytic functions and recyclability of the biocatalyst, enzyme immobilization is thought to be one of the strategies to overcome these problems [7,8].

There are various methods adopted for enzyme immobilization. The most common, which is of great interest, is by covalent bonds. It has been shown that the formation of covalent bonds between the enzymes and the active groups of the support material (-NH_2_, -COOH) prevents the enzyme from leaching out from the carrier and improves the reusability of the enzyme [9,10]. At the same time, the reaction approach could influence the enzyme activity. One of the most used methods in enzyme immobilization through covalent bonds involves the use of multifunctional reagents such as glutaraldehyde (GA) [11] with reactive ends that target specific functional groups on enzymes. Even if this method provides better mechanical properties and stability, the toxicity of such reagents, and challenges associated with their removal from finished products are the main limiting factors [12]. Moreover, since GA can react with internal amino acid residues involved in catalytic activity, it is not recommended for use with all enzymes [13]. The use of naturally occurring heterocyclic compounds obtained from plants, such as genipin, which is less toxic than GA, has also been documented. Chitosan-coated Fe_3_O_4_ NPs were effectively prepared by Gracida et al. [14], and genipin was used to crosslink them to xylanase. The immobilized xylanase showed improved stability under thermal and pH conditions, showing that the crosslinking process mediated by genipin can provide a more efficient conformational stabilization of enzymes. In another study, Hong et al. utilized genipin to prepare cross-linked enzyme aggregates (CLEAs) of *Trametes versicolor* laccase [13]. The new nanoconjugates showed great thermal and pH stability and very good activity even after 10 cycles of reuse (85%). However, genipin has some disadvantages that can make the replacement of GA or other similar compounds with increased cytotoxicity very difficult, including longer crosslinking time, high cost, separation problems of the unreacted compound and reduced available sources [15]. Over the years, many other compounds have been tested for enzyme immobilization, for example, epichlorohydrin [16] for chitosan-based biomaterials or dialdehydes [17], isocyanates [18], and carbodiimides [19] for collagen-containing systems, but they were often regarded as not so friendly agents for biomedical applications and may interact with bioligands, which would lessen the effectiveness of the immobilization process [20]. Therefore, a better approach or another multifunctional reagent with enough available sources and good biocompatibility for catalytic enzyme immobilization for inorganic/organic support are still necessary.

Glucose oxidase (GOx), an aerobic dehydrogenase, with a catalytic ability to generate gluconic acid and H_2_O_2_ by oxidation of β-d-glucose has been widely used over the years in the pharmaceuticals industry, in food technology or in manufacturing glucose biosensors [21,22]. However, in order to preserve their activity intact in the harsh conditions of food processing or biosensor manufacturing, various materials have recently been used for enzyme immobilization, such as carbon nanotubes [23], MnO_2_ nanosheets [24], iron oxides [25], mesoporous silica [26], polyacrylamide [27], polymethacrylates [28] and polysaccharides [29]. There is still a need for more effective and reusable supports despite the many GOx immobilization strategies that have been published over the past 10 years.

With a typically high load of ligands, MNPs are frequently utilized as supports for the physical and chemical immobilization of biomolecules. Additionally, magnetic decantation can be used to extract magnetic particles from the reaction mixture, lowering production costs. Due to their biocompatibility, low toxicity, advantageous magnetic properties and lack of retention of residual magnetism, superparamagnetic nanoparticles of Fe_3_O_4_ are the most widely used iron oxides among all varieties. However, Fe_3_O_4_ nanoparticles require stabilization and surface modification since, in addition to being rapidly air oxidized and losing their magnetic property, they have poor colloidal stability, particularly in neutral pH.

For immobilization of GOx Fe_3_O_4_ nanoparticles through their many unique features such as a large surface-to-volume ratio, high surface reaction activity and strong superparamagnetic behavior have proven to be a significant alternative to improve stability and recovery of the biocatalyst in many applications [30,31,32]. In addition, along with providing strong mechanical resistance and thermal stability, it prevents the loss of particles and increases the material’s resistance to degradation.

In the case of immobilization of GOx on the surface of magnetic nanoparticles (MNPs), the methods of coating nanoparticles with natural or synthetic polymers are preferred [33,34,35]. However, a suitable functionalization reaction of uncoated MNPs’ surface provides better active sites for enzyme attachment and improves their binding performance. Through surface silanization (3-aminopropyl triethoxysilane (APTES)), the surface of inorganic carriers is enriched with functional amino groups [36] that can be easily modified using carbonyl or thiol group-containing reagents. As mentioned above, the most used compound in such reactions is GA, a bi-functional reagent with aldehyde groups that is prone to react with GOx and immobilized it on MNPs through Schiff base formation. However, the toxicity and the increased concern regarding cell apoptosis of GA-crosslinked materials [37] has led to the search for another compound with the same functionality.

One such compound is 3,4-dihydroxy-3-cyclobutene-1,2-dione, known as squaric acid (SA) [38]. It has a cyclic structure and was utilized as an effective crosslinker for MNPs coated with chitosan/collagen [19], and collagen/elastin hydrogels [39] or in preparing a copolymacrolactone for essential oil encapsulation [40,41]. The influence of SA on the cytotoxicity of collagen/elastin hydrogels was assessed, revealing that SA can be regarded as a safe option [39] to replace the readily available reagents as GA for enzyme immobilization on amino-functionalized MNPs.

Therefore, the main goal of this study was to examine the possibility of replacing GA, a crosslinking agent which is frequently used to immobilize enzymes on MNPs, with another biocompatible compound with the same functionality, namely SA. To the best of our knowledge, there is no indication of any paper reporting on the utilization of SA as reagent for GOx immobilization on the surface of modified MNPs via APTES silanization.

In order to establish the effects of SA utilization on MNP stability before and after enzyme immobilization and to certify that indeed SA is a more suitable reagent for this kind of reaction, a comparison with GOx attached on nanoparticles using GA was also realized. A comparison of the structural and morphological changes after addition of SA or GA was made through Fourier-transform infrared spectroscopy (FT-IR) and scanning electron microscopy (SEM). The thermal stability and magnetic properties of the SA/GA-functionalized nanoparticles before and after GOx attachment were also investigated. The particle size distribution data obtained through dynamic light scattering (DLS) measurements showed that the formed particles were in the nanometric range. Moreover, after establishing the optimal synthesis parameters for a good colloidal stability of GOx-coated MNP, the efficiency of enzyme immobilization and the remanent activity of GOx were determined. The obtained results clearly show that SA is a good candidate for GOx immobilization on pristine MNPs with potential application in the biomedical field.

## 2. Materials and Methods

### 2.1. Materials

Glucose oxidase (EC 1.1.3.4) type II-S from Aspergillus niger (activity 18.2 U/mg), glucose, squaric acid, glutaraldehyde, 3-(aminopropyl)-triethoxysilane (APTES, 99%), 4-aminoantipyrine (4-AAP), phenol, peroxidase from horseradish Type I (activity ≥50 units/mg solid) and Coomassie Brilliant Blue G-250 for protein assay used in this work were purchased from Sigma Aldrich (Hamburg, Germany). Ferric chloride hexahydrate (FeCl_3_·6H_2_O) and ferrous chloride tetrahydrate (FeCl_2_·4H_2_O) were obtained from Merck (Darmstadt, Germany).

All aqueous solutions were prepared with deionized water that had been passed through a Millipore Milli-Q Plus water-purification system. All chemicals were of analytical grade and used as received.

### 2.2. Synthesis and Surface Modification of Fe_3_O_4_ MNPs

In the initial stage, Fe_3_O_4_ MNPs were synthesized using the conventional co-precipitation method, described in the literature [42]. A mixture of FeCl_3_ × 6H_2_O and FeCl_2_ × 7H_2_O (*n*(Fe^3+^):*n*(Fe^2+^) = 2:1)was dissolved in 100 mL of ultrapure water in the presence of N_2_. The solution was stirred at 60 °C for 3 h in an inert atmosphere. The pH of the solution was adjusted to 10.0 by the dropwise addition of a 5.0 M NaOH solution. Subsequently, the precipitate formed was separated from the reaction mixture by an external permanent magnet, washed with ultrapure water and ethanol three times and resuspended at a final concentration of 50 mg/mL.

In the second stage, the surface of Fe_3_O_4_ MNPs was modified through silanization with (3-aminopropyl) triethoxysilanes (APTES) to obtain amino-functionalized nanoparticle. According to the literature, the optimal molar ratio of surface modification of MNPs with APTES is 4:1. Therefore, for this reaction, 0.8 mL of APTES was added under an inert atmosphere over 80 mL of ethanol/water solution (1:1 *v*/*v*) of MNPs (0.2 g). After stirring at 40 °C for 24 h, the MNPs coated with amino groups were separated on the permanent magnet, washed three times with ultrapure water and ethanol, and then dried in a vacuum at 70 °C for further characterization.

### 2.3. Immobilization of Glucose Oxidase on APTES-Modified MNPs

APTES-modified MNPs (AMNPs) were reacted with two bifunctional compounds with carbonyl groups: glutaraldehyde (GA) and squaric acid (SA). The solution of MNPs modified with APTES (0.2%) was mixed with an aqueous solution of SA/GA of various concentrations after being properly sonicated, and the obtained mixtures were left under constant stirring at 37 ± 2 °C for 24 h. The nanoparticles were separated on a semi-permanent magnet and washed with ultrapure water, and then used in the final stage to immobilize GOx.

Immobilization was achieved by the following approach: GOx dissolved in buffer solution (0.01 M, pH 6.5) was mixed with AMNPs nanoparticles in various ratios and placed in a shaker incubator at room temperature for 6 h. The nanoparticles were washed with the same buffer solution to remove free GOx.

**Figure 1:**
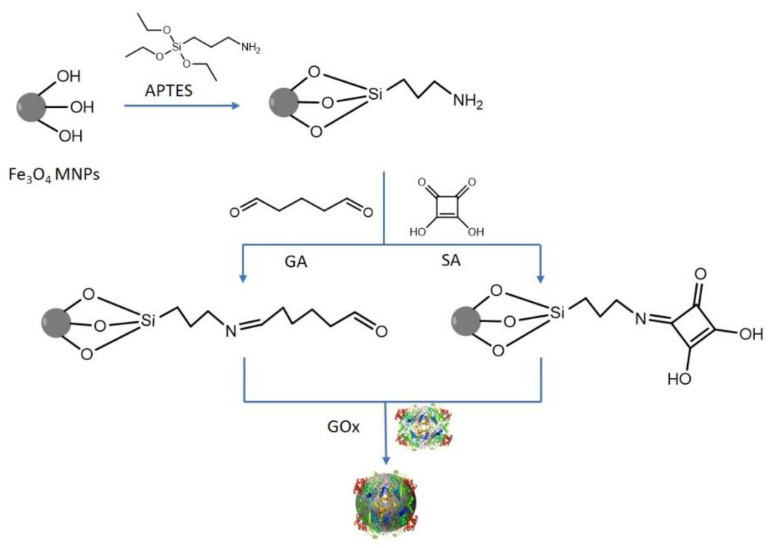
Representative scheme for MNPs functionalization and GOx coupling.

The immobilization technique shown in Figure 1 was based on using GA or SA as coupling agents to link the GOx enzyme covalently to the MNPs that had previously been functionalized with APTES. GA/SA forms imine bonds with the amino group from APTES-modified MNPs, while the free terminal aldehyde group reacts with the amino residues of the enzyme. Data analysis and graphing were performed with Origin 2018 SR1.

### 2.4. Dynamic Light Scattering (DLS) Measurements

Particle-size distribution, polydispersity (PDI) and zeta potential (ZP) of the functionalized Fe_3_O_4_ nanoparticles and those with GOx were investigated in aqueous solutions using DLS. ZP is defined as the electric potential of the nanoparticles measured in the diffuse layer around their surface, which is also called the shear plane [43]. It is an important parameter as ZP values are highly correlated with particle shape and colloidal stability. The samples were prepared in distilled water and ultrasonicated at room temperature before measurements. DLS analyses were carried out with Malvern Nano-ZS (Malvern Instruments, Worcestershire, UK) at 25 °C. The average of at least three measurements was taken. Data analysis was performed with the Zetasizer software provided by Malvern, and the graphing was performed with Origin 2018 SR1.

### 2.5. Morphological Characterization

The morphology of the freeze-dried samples was investigated by scanning electron microscopy (SEM; Quanta 200 with EDAX—Elemental Analysis System). The device was operated with secondary electrons at 20 kV, under low vacuum mode (60–100 Pa) and LFD detector. Samples were sputtered with gold before analysis. Data analysis was realized using the software ImageJ.

### 2.6. Fourier-Transform Infrared (FT-IR) Analysis

FT-IR spectra of precursors and GOx-immobilized nanoparticles were recorded using a Vertex Bruker Spectrometer in transmittance mode. Spectra were collected as an average of 64 scans at a resolution of 4 cm^−1^ within the range from 4000 to 400 cm^−1^ (KBr pellets technique). Data graphing was performed with Origin 2018 SR1.

### 2.7. Vibration Sample Magnetometer (VSM)

Magnetic measurements were realized using a Quantum Design-PPMSQD-9 vibrating sample magnetometer at room temperature under an applied magnetic field in the range of −30–30 kOe for M-H curves. Data analysis and graphing were performed using Origin 2018 SR1.

### 2.8. Thermogravimetric Analysis (TGA)

Thermogravimetric analysis was carried out on dried nanoparticles using an online TG-DSC/FT-IR/MS system. The system is equipped with an apparatus of simultaneous thermogravimetric and differential scanning calorimetry analyses model STA 449F1 Jupiter (Netzsch, Selb, Germany), FT-IR spectrophotometer Vertex-70 model (Bruker-Germany) and mass spectrometer QMS 403C Aëolos model (Netzsch, Selb, Germany). The samples were heated under a nitrogen flow with a 50 mL/min^−1^ rate in an open Al_2_O_3_ crucible. Analyses were performed in dynamic mode at a heating rate of 10 °C/min from room temperature up to 600 °C. Data collection was realized with Proteus^®^ software.

### 2.9. Assay of GOx Activity

The activity of free GOx and GOx-coated nanoparticles was estimated by monitoring the oxidation of glucose according to the Trinder colorimetric method reported in the literature, with some modifications [44]. An assay mixture was prepared by mixing 500 U of horseradish peroxidase, 0.015 mmol of 4-aminoantipyrine (4-AAP), 0.025 mmol of phenol and 5 mmol of glucose in 50 mL of phosphate buffer solution (0.1 M, pH 7.0). To start the enzymatic reaction, 2 mL of the assay solution was added to glass vials containing GOx-coated nanoparticles and vigorously stirred for 30 s at room temperature. A solution of free GOx of the same molar concentration was used to evaluate the activity of the free enzyme for comparison. Next, the supernatant was separated from the MNPs using a semi-permanent magnet; then, aliquots of the supernatant were taken and the absorbance was read at 500 nm to determine the concentration of H_2_O_2_ [44]. Data analysis and graphing were realized with Origin 2018 SR1.

The activity of the enzyme can be calculated using Equation (1) [44]:

(1) $$Concentration\;of\;glucose\;\left(\frac{\mathrm{mg}}{L}\right)=\frac{Abs\left(t\right)}{Abs\left(s\right)}*C\left(s\right)\;\left(\frac{\mathrm{mg}}{L}\right)$$

where *Abs*(*t*) is the absorbance value of the sample, *Abs*(*s*) is the absorbance value of the standard solution and *C*(*s*) is the concentration of the standard solution.

The enzyme activity was determined in triplicate. One unit of enzyme activity was defined as the amount of enzyme that oxidized 1 μmol substrate (glucose) to end-product per min at 25 °C.

### 2.10. Immobilization Efficiency Determination (Bradford Protein Assay)

The amount of immobilized GOx on MNPs in relation to the time reaction was determined using the Bradford protein assay. For determination of immobilized GOx concentration, about 100 µL of the supernatant (containing unbound GOx after magnetic separation of the nanoparticles) was mixed with the dye reagent concentrate. After 5 min, the absorbance of the dye solution was measured at 595 nm. A standard curve was realized to establish the immobilization efficiency of the enzyme. The immobilization efficiency was calculated based on Equation (2) [45]:

(2) $$Immobilization\;efficiency\;\left(\%\right)=100-\left(\frac{Abs_{unbound\;GOx}}{Abs_{free\;GOx}}\right)\times 100$$

where *Abs_unbound GOx_* is the absorbance value of the unbound GOx from the supernatant and *Abs_free GOx_* is the absorbance value of the free GOx from the supernatant without nanoparticles. Data analysis and graphing were performed with Microsoft Excel 2016.

## 3. Results and Discussion

Immobilized enzymes have important advantages over soluble enzymes or other alternative technologies such as higher stability and ease of removal from the reaction mixture. Moreover, in nanotechnology, the immobilization of biocatalysts such as glucose oxidase or laccase on organic or inorganic substrates are very advantageous, leading to the development of nanodevices with multifunctionality, for example, sensing or separation. However, for the immobilization of an enzyme on a support, it is important to choose the most suitable coupling agent in order to preserve its catalytic activity. This study aimed to investigate the possibility of replacing one of the most widely used compounds for immobilizing enzymes on MNPs, namely GA, with another bifunctional and biocompatible compound such as SA. Using a comparative study, the colloidal stability of the new MNPs functionalized with SA or GA in buffer solutions that simulate a favorable environment for enzyme activity (GOx) was investigated, as well as the remaining catalytic activity.

### 3.1. DLS Investigations

#### 3.1.1. DLS Measurements Regarding the Stability of GA/SA-Functionalized Magnetic Particles without Enzyme

The optimal conditions for the process of GOx immobilization on the surface of MNPs were established after determining the hydrodynamic diameter (D_h_) and ZP of the functionalized and enzyme-conjugated MNPs.

Studies were performed on the stability of MNPs after surface silanization with APTES (AMNP) and coupling GA or SA. Initially, it was determined that the optimal concentration of reagent (GA or SA) does not alter the stability of magnetic particles in physiological conditions (see Table 1 for the used ratios between the compounds). Then, in the second stage, after establishing the optimum concentration of the functionalizing agent (GA or SA), the stability of the nanoparticles conjugated with GOx was investigated by varying the ratio between the enzyme and the particles as well as the immobilization time (see Table 2).

By comparison with bare MNPs and amino-functionalized MNPs (AMNPs), the AMNP_GA nanoparticles presented lower D_h_ when the used GA concentrations were in the 1.0–3.0% range. Furthermore, MNPs functionalized with GA presenting the smallest diameters were reported by Banerjee and Chen, with GA being added after pristine MNP synthesis through a sonication procedure [46], exactly as in our study.

**Figure 2:**
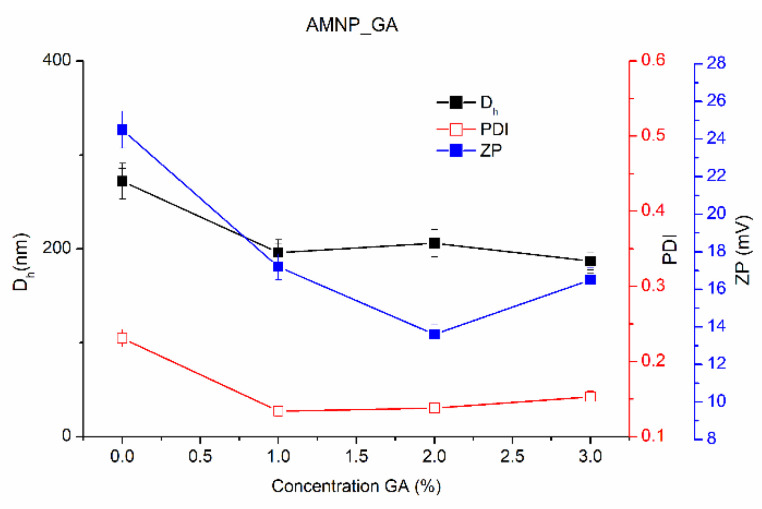
Variation of D_h_, PDI and ZP with the GA concentration used to functionalize the MNPs.

In the case of GA-functionalized particles, the ZP values slightly decrease with increasing GA concentrations (AMNP_GA), from +17.2 mV to approximately +14.6 mV (Table 2 and Figure 2). After APTES functionalization, the ZP changes to positive proportionally with the new created amino groups on the nanoparticles’ surface. When GA is used, the ZP remains positive but slightly decreases, indicating the creation of new imine bonds, but also that some amino groups remain free. In order to ensure that all the amino groups reacted, an increase in the GA concentration is needed, that will negatively influence the cytotoxicity of the nanoparticles. These observations are consistent with those of Mariño et al. [47].

Moreover, the colloidal stability of AMNP_GA nanoparticles over time (1 week) is ensured even when ZP values are decreasing by comparison with the AMNP nanoparticles (+24.5) (data not presented in the material). This evolution of the surface properties confirms the increase in GA-functionalization density following the reaction between the amino groups of APTES moieties on the surface of MNP and GA. The reaction with GA is also confirmed by the decrease in ZP of AMNP nanoparticles from +24.5 mV to +14.6 mV, values obtained before and after adding GA.

Furthermore, after treating the surface of the amino-functionalized MNPs with SA, the new AMNP_SA particles showed a ZP value depending on the concentration of SA, similar to the case of GA functionalization. SA-modified nanoparticles showed a ZP value that decreased from −13.6 to −16.9 with increasing SA concentration from 0.25 to 1%, attesting SA coupling. The D_h_ decreased with increasing SA concentration from 179 (AMNP_SA0.25) to 154 nm (AMNP_SA0.5) while maintaining a PDI above 0.2, thus demonstrating an increased stability of SA-functionalized AMNPs.

**Figure 3:**
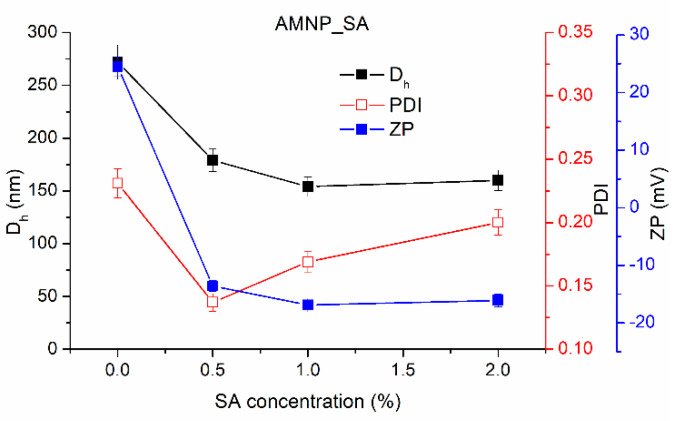
Variation of D_h_, PDI and ZP with the SA concentration used in the functionalization of MNPs.

In the case of SA functionalization, at even low concentration, the negative values of the ZP indicates a deprotonation of the SA hydroxyl groups, which may show that all the amino groups were reacted (Figure 3 and Table 1).

However, despite the low surface ZP in aqueous medium (pH = 6.0) that can lead to formation of agglomerated clusters of the MNPs, the aggregation is practically negligible, with D_h_ ranging from 154 to 179 nm for AMNP_SA and between 187 and 206 nm for AMNP_GA (Figure 2 and Figure 3). It is probable that the ethyl groups of APTES and the functionalizing agents SA and GA ensure the steric balance by generating an electrostatically stabilized system with a proportionally higher stability. Compared to the nanoparticles’ stability, which is only generated by electrostatic repulsive forces of the chemically attached protonated amino groups on the surface of MNPs, the new synthesized nanosystem has a proper functionalized surface that will allow a better conjugation of GOx.

For a comparative study of GOx immobilization on GA/SA-functionalized MNPs, the variants AMNP_GA1 and AMNP_SA0.5 were selected because those batches presented the best colloidal stability (see Table 1).

#### 3.1.2. DLS Measurements Regarding the Stability of GA/SA-Functionalized Magnetic Particles Conjugated with GOx

##### Effect of SA/GA-Functionalized Nanoparticles/Enzyme Ratio on the Nanoparticles’ Stability

Because it is mentioned in the literature that the optimal activity of GOx in immobilization reactions on different substrates is in the pH range of 6.0–7.0, the stability of MNPs functionalized with GA or SA and conjugated with GOx was studied at pH 6.5 [48]. It is important to mention that as the DLS measurements were performed in PBS, pH 6.5, the obtained values (D_h_, PDI and ZP) for AMNP_GA1 and AMNP_SA0.5 will be different as compared to those listed in Table 1.

**Figure 4:**
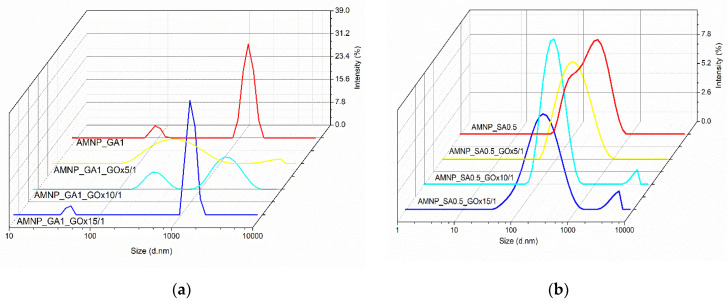
Size distribution reported to the nanoparticles/enzyme mass ratio (**a**) AMNP_GA1 and (**b**) AMNP_SA0.5 in buffer, 0.01 M, pH 6.5 and room temperature.

Different mass ratios between AMNP_GA/SA and GOx were also evaluated (Figure 4), keeping the AMNP_GA/SA concentration (2 mg/mL) and immobilization time (6 h) constants and using the variant of AMNP_SA/GA with the optimal colloidal stability previously determined, namely AMNP_GA1 and AMNP_SA0.5. The AMNP_GA/SA variants with low concentrations of functionalization agents (GA or SA) were chosen in order to discard any factors that may influence the activity site mobility of the enzyme which in turn can lead to a decrease in the immobilization yield. In addition, in the literature, it is mentioned that a higher concentration of GA might affect the tridimensional structure of various enzymes such as laccases following immobilization reaction [49,50].

According to the results data presented in Figure 4, at an AMNP_GA1/enzyme mass ratio of 5/1, a good stability of GA-functionalized MNPs and those conjugated with GOx was observed compared to the other used ratios (10/1 and 15/1, respectively). By decreasing the amount of enzyme, Dh and PDI values of the AMNP_GA1_GOx10/1 and AMNP_GA1_GOx15/1 systems increased, indicating a tendency toward instability in buffer solution, pH 6.5. There was also a slight decrease in the ZP from −18.3 mV to −18.8 mV which may be due to the decrease in the enzyme ratio used in the immobilization reaction. AMNP_GA1_GOx15/1 particles showed a dimensional distribution similar to that of AMNP_GA1 without the enzyme that changes when more GOx is added and tends to have slightly better colloidal stability. Thus, it is confirmed that in the case of AMNP_GA1 nanoparticles, at pH 6.5, by increasing the enzyme ratio, several enzyme molecules are adsorbed onto the active sites on the surface of the MNPs, inducing a better colloidal stability.

By comparison, the MNPs functionalized with 0.5% SA showed in the pH 6.5 buffer solution a negative ZP value of −30.6 mV. By adding GOx, an increase in the ZP value was observed: from −29.9 mV for AMNP_SA0.5_GOx15/1 to −25.6 mV for AMNP_SA0.5_GOx5/1. Moreover, a decrease in particle size and PDI is obvious, which indicates a higher colloidal stability for AMNP_SA0.5 nanoparticles conjugated with GOx due to the combined steric and electrostatic effects. By analyzing the data presented in Figure 4, we found that GA-functionalized particles showed good stability at 5/1 AMNP_GA1/GOx mass ratio, but were characterized by two particle populations (327 and 1971 nm), indicating a possible agglomeration over time (6 h). Instead, AMNP_SA0.5 particles were monodispersed for the entire period of the measurements, with a peak at 221 nm.

Therefore, the overall results implied that after functionalization of MNP surface with SA and immobilization of GOx, the stability of the produced nanoparticles was improved, which can be attributed to the increase of hydrophilicity after enzyme binding to the MNPs. This observation has been made by other researchers in regard to the obtained magnetic bionanoconjugates [51].

##### Effect of Immobilization Reaction Time on the Stability of GOx-Conjugated MNPs

The period of immobilization time was also evaluated due to its influence on the Gox attachment and possibly on the enzyme conformation change upon immobilization, as different papers reported a maximum activity of the enzyme in a specific time interval with a decrease after this period [48]. Some of the possible reasons during the process of immobilization are: conformation change or denaturation of the enzyme [52], occurrence of supplementary new bonds between enzyme and support, or aggregation of the support.

**Figure 5:**
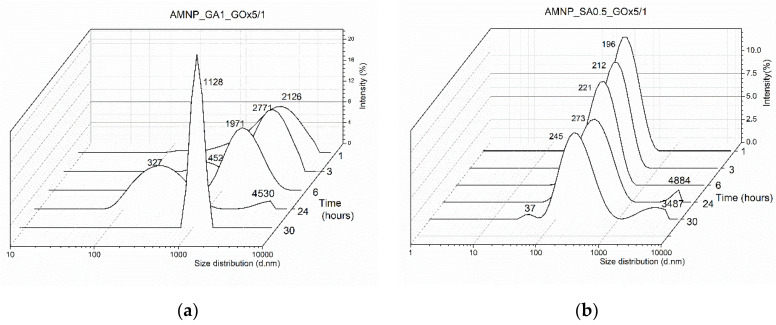
Size distribution reported to the GOx immobilization time of the nanoparticles: (**a**) AMNP_GA1 and (**b**) AMNP_SA0.5 in buffer solution, 0.01 M, pH 6.5 at room temperature.

Although in the case of particles functionalized with SA, the increase of the immobilization time led to the slight reduction in colloidal stability due to the supersaturation of the active sites on the AMNP_SA0.5 surface destined to the enzyme attachment (Figure 5), in the case of particles with GA, the colloidal stability improved after 24 h [53] but again decreased after 30 h. Naturally, longer periods of immobilization result in higher enzyme immobilization of the nanoparticles, allowing a prolonged interaction between enzyme molecules and particle surface.

However, for AMNP_SA0.5 particles, by increasing the contact time to over 6 h, the enzyme is most likely deactivated due to the high frequency of collision with the enzyme molecules already attached to the surface of the magnetic particle, and finally, the immobilization is reduced. As can be seen in Figure 5a,b, a better stability was obtained after 6 h for SA-functionalized MNPs and at 24 h for GA-functionalized particles. In aqueous conditions, a good colloidal stability avoids the development of nanoparticle aggregates, which prevents the loss in biocatalytic efficacy of the enzyme following redispersion of the nanocarrier. As can be seen from the results, SA-functionalized nanoparticles conjugated with GOx have a better colloidal stability over time, even after 30 h, as compared with the MNPs modified with GA. Moreover, the fact that the immobilization of the enzyme takes place in a shorter time, as in the case of AMNP_SA0.5 nanoparticles, can contribute substantially to the prevention of enzyme degradation and to the preservation of its catalytic activity.

### 3.2. Scanning Electron Microscopy (SEM)

**Figure 6:**
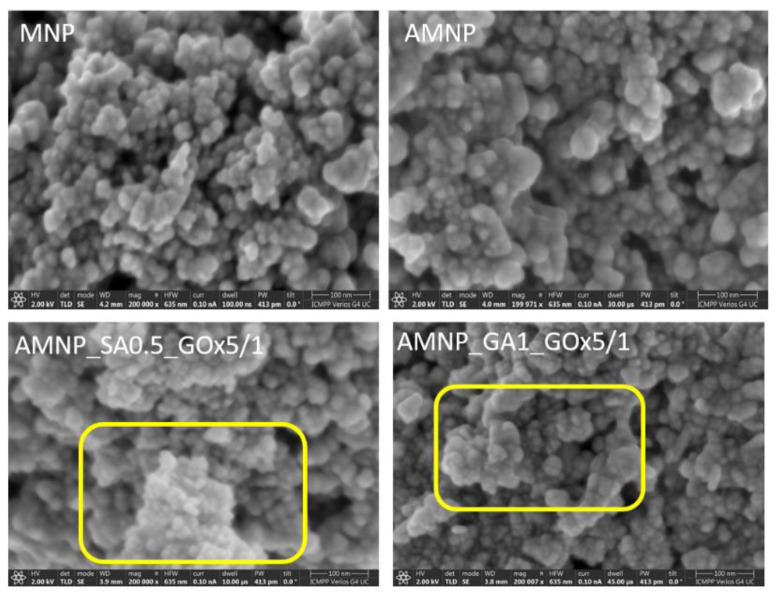
Optical microscopy data for MNPs, AMNPs and enzyme-conjugated MNPs.

The size and morphology of pure MPNs, those protected with APTES and functionalized with GA or SA as well as those conjugated with GOx were investigated by SEM (Figure 6). It can be seen from the SEM images of unmodified MNPs that most of the particles were in the form of agglomerated spherical structures. This could be due to the coercive force of the MNPs and the magnetic dipolar interaction among the nanoparticles as a result of their magnetic properties. Moreover, the unmodified MNPs are made up of clusters of agglomerated nanoparticles with dimensions between 10 and 25 nm. After the functionalization of nanoparticles with APTES (AMNP), the particle size of AMNP ranged from 30 to 45 nm. These findings are consistent with those of Xu et al. [54]. Compared to MNP, AMNPs have larger particle diameters and a greater uniformity of size distribution, which confirms that MNPs have been protected with APTES. AMNP_GA1_GOx5/1 (28–47 nm) and AMNP_SA0.5_GOx5/1 (25–40 nm) particles have a cluster conformation and are agglomerated, which may also be due to the attachment of the biological macromolecule, confirming the immobilization of GOx on the surface of functionalized MNPs (areas highlighted in the microscopy images below by boxes). The observations regarding the shape and morphology of the nanoparticles characterized in this study are similar with those made by Nadar et al. [55].

### 3.3. FT-IR Analysis

FT-IR spectra of GA/SA-unfunctionalized MNPs before and after GOx immobilized were recorded and compared to confirm surface functionalization and enzyme immobilization. Firstly, the spectra of MNPs functionalized with APTES and GA/SA and conjugated with GOx were analyzed. In the spectrum corresponding to nanoparticles protected with APTES and functionalized with GA (AMNP_GA1), the presence of peaks from 583 and 928 cm^−1^ characteristic of the Fe-O and Si-O-Fe bonds is observed, thus confirming the modified surface of the MNPs. The binding of GOx to the surface of magnetic Fe_3_O_4_ nanoparticles was also confirmed by FT-IR analysis. The absorption band that appears in the range 3400–3300 cm^−1^ was observed in all samples and is attributed to the overlapping stretching vibrations of the OH and NH bonds present on the surface of all nanosystems and pure GOx. These findings are consistent with those of Nematidil et al. [56].

**Figure 7:**
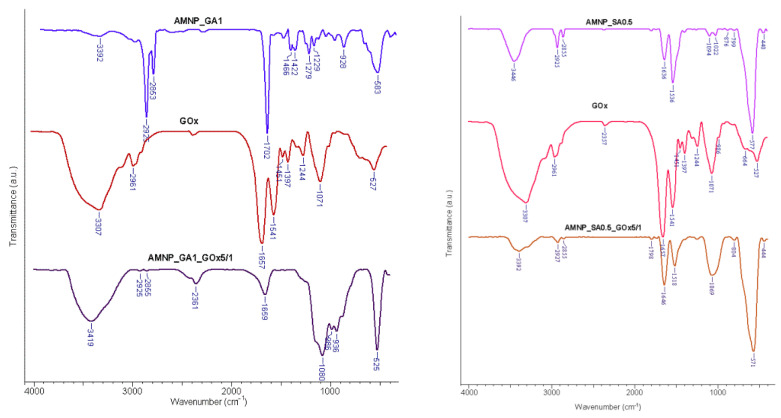
FT-IR spectra of AMNP_GA1 and AMNPSA0.5 before and after GOx immobilization.

In the case of the AMNP_SA0.5_GOx5/1 sample spectrum, the peak at 1646 cm^−1^ denotes the formation of C=N bonds as a result of the functionalization reaction of MNP nanoparticles with SA (Figure 7), and also present in the FT-IR spectrum of AMNP_GA1_GOx5/1 at 1659 cm^−1^. The bands at 1060 and 1069 cm^−1^ present in the spectra of AMNP_SA0.5_GOx5/1 and AMNP_GA1_GOx5/1, respectively, are attributed to the deformation vibration of the C=O bond found in the structure of GOx. Furthermore, the absorption band characteristic of Fe-O bond appears at 571 and 525 cm^−1^ in the spectra of AMNP_SA0.5_GOx5/1 and AMNP_GA1_GOx5/1, respectively. The appearance of bands characteristic of the enzyme on the magnetic bioconjugates spectra confirms the immobilization reaction of GOx on the SA/GA-modified surface of MNPs.

### 3.4. Magnetic Properties

**Figure 8:**
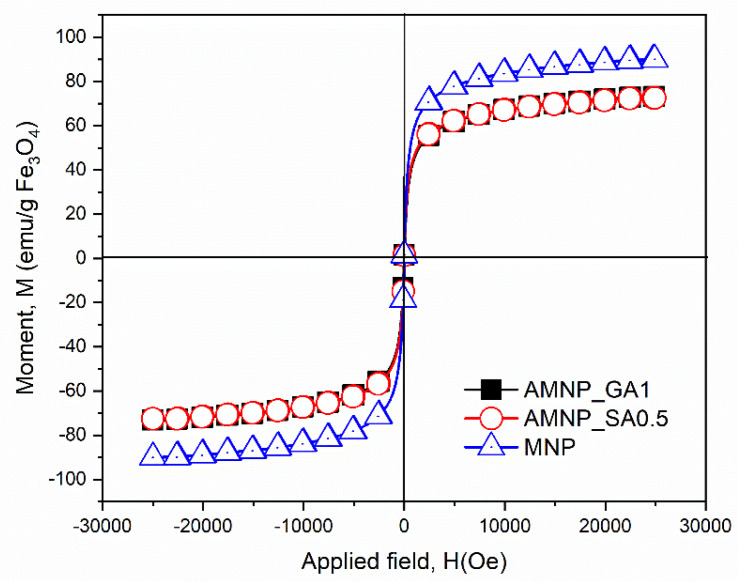
Magnetization curves for simple and GOx-coated MNPs.

The plots of the magnetization (M) versus magnetic field (H) at 25 °C for the nanoparticles with and without immobilized GOx are shown in Figure 8. From the M-H curves, the saturation magnetization (Ms) was determined and recorded [57].

Ms for MNPs was 89.9 emu. A gradual decrease in Ms was observed for nanoparticles AMNP_GA1_GOx5/1 and AMNP_SA0.5_GOx5/1. The Ms values for AMNP_GA1_GOx5/1 and AMNP_SA0.5_GOx5/1 were 72.55 and 73.18 emu, respectively, which are slightly smaller than the nanoparticles without enzyme. This decrease in Ms could be attributed to the enzyme coupling on the MNPs’ surface, which might quench the magnetic moment. According to the values obtained above, the AMNP_GA1_GOx5/1 and AMNP_SA0.5_GOx5/1 nanoparticles demonstrated properties attributed to a superparamagnetic material. These observations were consistent with those of Sohn et al. [57].

As mentioned above, studies have shown the effectiveness of using SA as a functionalizing agent for enzyme immobilization. Moreover, in our study, it was shown that compared to the classical agent (GA), there are clear improvements brought by SA functionalization on MNP properties: smaller particle size and better stability and similar superparamagnetic behavior. These corroborated data show that the variant AMNP_SA0.5_GOx5/1 is an optimal system. For these reasons, this variant will be chosen for subsequent characterizations regarding thermal stability, efficiency of GOx immobilization on MNPs and the residual catalytic activity of GOx after attachment of nanoparticles.

### 3.5. Thermal Characteristics

The thermal behavior of pristine MNPs, GOx and enzyme-conjugated AMNP_SA0.5 was investigated to gather additional information about their thermal stability changes as a result of GOx coupling to the nanoparticles’ surface.

**Figure 9:**
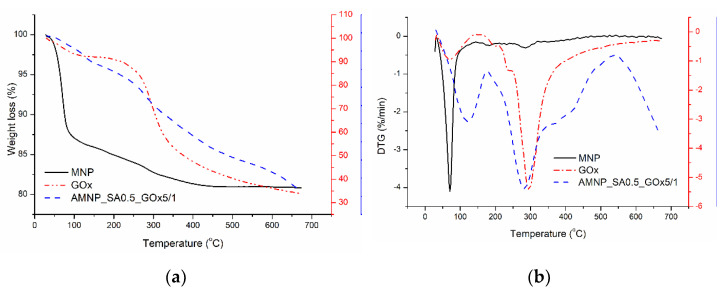
TG (**a**) and DTG (**b**) graphs corresponding to MNPs, enzyme (GOx) and amino-functionalized MNPs conjugated with enzyme via SA (AMNP_SA0.5_GOx5/1).

Therefore, Figure 9 shows the TG and DTG curves of the MNPs, GOx and AMNP_SA0.5_GOx5/1 samples, indicating differentiated weight loss that occurred as a result of not only the increase in temperature but also the composition of the analyzed systems. All samples were characterized by weight loss with a T_peak_ at 70.6 (MNP), 69.5 (GOx) and 127.9 °C (AMNP_SA0.5_GOx5/1) due to the elimination of the physically absorbed water (Table 3). In the case of AMNP_SA0.5_GOx5/1 nanoparticles, the second stage of the thermal degradation process characterized by a mass loss of 13.3% and a T_peak_ at 287.9 °C was attributed to the degradation of the enzyme, with the release of carboxyl and amino groups contained in GOx. This stage is also found in the degradation process of the enzyme, being representative of stage IV (Table 3) and for which a mass loss of 51.9% was recorded. As a result, the changes in the percentage of weight loss of enzyme-coated MNPs as compared with the pristine MNPs indicated immobilization of GOx on the surface of the nanoparticles.

### 3.6. Efficiency of Enzyme Immobilization (Bradford Assay)

The immobilization of enzymes in nanoparticles usually depends on many physical and chemical factors such as the immobilization time, concentration of nanoparticles, degree of functionalization of the nanoparticles’ surface, reaction temperature, immobilization time and buffer solution pH levels [58,59]. Therefore, in our study, it was important to investigate the efficiency of GOx immobilization for AMNP_SA0.5 nanoparticles with respect to the immobilization time.

**Figure 10:**
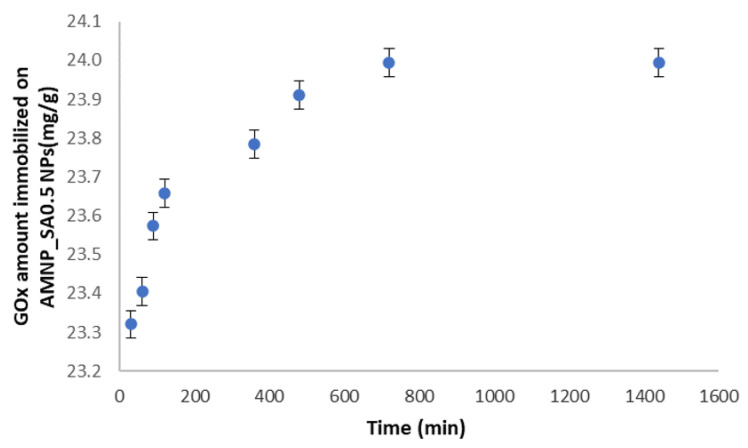
Variation in the amount of enzyme immobilized on AMNP_SA0.5 nanoparticles’ surface (mg/g MNPs) over time.

The amount of immobilized enzyme was determined by the Bradford method. As shown in Figure 10, the amount of immobilized enzyme gradually increased with time of immobilization from 30 min to 8 h, after which it became almost constant (24 h). Therefore, the maximum amount of immobilized enzyme was 23.91 mg/g of AMNP_SA0.5 nanoparticles after 24 h; this observation indicates that the amino groups of GOx on the MNP surface functionalized with SA can saturate (block) the specific sites for enzyme coupling in the first 6 h, and the reaction continues until a plateau is reached. These conditions ensure a homogeneous functionalization among the population of AMNPs functionalized with SA. Therefore, in accordance with the results from the Bradford assay, the optimal immobilization time of the enzyme on MNP was estimated to be around 8 h after the start of the reaction. The binding mechanism of GOx to the AMNP_SA0.5 surface was confirmed from the FT-IR spectrum and might be influenced by the structure of the GOx enzymatic macromolecule.

In conclusion, through the binding reaction between GOx and amino-functionalized MNPs via SA, the stability of the enzyme against chemical and mechanical stress can be improved.

### 3.7. Evaluation of the Activity of Enzyme-Conjugated Nanoparticles

The activity of the GOx enzyme was determined using a colorimetric method following the Trinder procedure [11] based on the measurement of the initial rate of hydrogen peroxide formation at 25 °C, which results in a dye: quinone red. The principle of enzymatic determination of GOx activity is described as follows: Glucose is oxidized by GOx to gluconate and hydrogen peroxide. Phenol and 4-aminoantipyrine, in the presence of peroxidase, produce a quinone-imine dye which is measured at 500 nm using a UV-VIS spectrophotometer. Thus, absorbance values proportional to the glucose concentration in the samples are obtained (Equation (1)).

Three mass ratios of AMNP_SA0.5, 15:1, 10:1 and 5:1 (corresponding to an enzyme concentration of 4.8, 9.6 and 19.2 U per mg of AMNP_SA0.5 nanoparticles, respectively), were used to determine residual activity of the enzyme on the surface of AMNP_SA0.5 nanoparticles. The activity of the immobilized enzyme was: 0.008, 0.032 and 0.1 U/mg AMNP_SA0.5 for the ratios 15:1, 10:1 and 5:1, respectively.

Therefore, a higher concentration of the enzyme led to an increase in the enzyme bound per unit mass of MNP. With a higher amount of AMNP_SA0.5, the amount of immobilized enzyme was lower, indicating that the main limitations may be the concentration of the enzyme or the competition for coupling with other residual proteins left in the process of obtaining GOx. A direct correlation between enzymatic activity and the amount of AMNP_SA0.5 cannot be estimated, as only those enzymes that are coupled to the surface and are still active can give positive results when using the Trinder colorimetric method. During immobilization, the enzyme covalently binds through its free amino terminal groups to the carbonyl groups of SA.

Immobilized GOx may also have reduced enzyme activity with respect to soluble enzymes, due to conformational changes that can occur during enzyme coupling onto inorganic substrate.

## 4. Conclusions

In this study, glucose oxidase (GOx) was covalently immobilized on the surface of modified iron oxide MNPs using a new and safer reagent, namely SA. In order to establish the effects of SA surface functionalization on MNP stability before and after enzyme coupling and to demonstrate that SA is indeed a more suitable reagent for this kind of reaction, a comparison with GOx attached onto GA-modified nanoparticles was also realized.

The particle size and colloidal stability characterization of the pristine/surface functionalized MNPs and of those coupled with GOx were characterized, emphasizing the difference between the two functionalization reagents. Binding of GOx to MNPs by using GA or SA was confirmed by FT-IR spectroscopy. The particle size distribution data obtained through DLS measurements showed that the formed particles were in the nanometric range.

A suitable colloidal stability was obtained after 6 h for SA-functionalized MNPs and at 24 h for GA-functionalized particles. Moreover, the fact that the immobilization of the enzyme takes place in a shorter time, as in the case of AMNP_SA0.5 nanoparticles, can contribute substantially to the prevention of enzyme degradation and to the preservation of its catalytic activity. Compared to MNP, AMNPs had larger particle diameters and a greater uniformity of size distribution. AMNP_GA1_GOx5/1 (28–47 nm) and AMNP_SA0.5_GOx5/1 (25–40 nm) particles had a cluster conformation, which may be due to the attachment of the biological macromolecule. Regarding the magnetic properties, Ms values for AMNP_GA1_GOx5/1 and AMNP_SA0.5_GOx5/1 were 72.55 and 73.18 emu, respectively, which were slightly smaller than the nanoparticles without enzyme, thus confirming the enzyme coupling on the MNPs surface and the superparamagnetic behavior of the GOx-immobilized MNPs.

As the variant AMNP_SA0.5_GOx5/1 was chosen as the optimal system, the analysis of GOx immobilization efficiency and thermal degradation analysis were performed only on AMNP_SA0.5 nanoparticles. Thus, GOx immobilization efficiency with respect to the immobilization time revealed that the optimal immobilization time of the enzyme on MNPs was estimated to be around 8 h after the start of the reaction, with a maximum amount of immobilized enzyme of 23.91 mg/g of AMNP_SA0.5 nanoparticles.

As compared with the simple enzyme, AMNP_SA0.5_GOx5/1 nanoparticles had a thermal degradation process characterized by two stages instead of four that corresponded to the elimination of bound water and to the release of carboxyl and amino groups contained by the immobilized GOx. The last stage is also found in the degradation process of the enzyme, being representative of stage IV with a mass loss of 51.9% in comparison with 13.3% for the second stage of AMNP_SA0.5_GOx5/1 thermal degradation. As a result, the changes in the percentage of weight loss of enzyme-coated MNPs as compared with the pristine GOx and unmodified MNPs indicated immobilization of GOx on the surface of the nanoparticles.

The obtained results clearly show that SA is a good candidate to fully replace GA, a crosslinker with proven cytotoxicity and major problems regarding material purification for GOx immobilization on MNPs. Moreover, these new SA-bioconjugates can be regarded as a catalytic nanodevice for biomedical purposes such as blood glucose monitorization.

## Figures and Tables

**Table 1:** Characteristics obtained in the DLS determinations for pristine MNPs, AMNPs and those functionalized with GA/SA.

Sample	GA/SA Concentration (%)	D_h_ (nm)	PDI	ZP (mV)
MNP	-	206 ± 5.18	0.186 ± 0.005	−10.9
AMNP	-	272 ± 6.16	0.231 ± 0.007	+24.5
AMNP_GA1	1	196 ± 4.88	0.134 ± 0.004	+17.2
AMNP_GA2	2	206 ± 4.26	0.138 ± 0.003	+14.6
AMNP_GA3	3	187 ± 5.61	0.153 ± 0.005	+16.5
AMNP_SA0.25	0.25	179 ± 6.47	0.137 ± 0.004	−13.6
AMNP_SA0.5	0.5	154 ± 4.62	0.169 ± 0.005	−16.9
AMNP_SA1	1	160 ± 7.80	0.2 ± 0.006	−16.1

**Table 2:** Characteristics obtained in the DLS measurements for AMNP_GA/SA conjugated with GOx depending on the nanoparticle/enzyme ratio in buffer solution with pH 6.5.

Sample	Nanoparticles/Enzyme Ratio (*w*/*w*)	D_h_ (nm)	PDI	ZP (mV)
AMNP_GA1	-	2867 ± 83.01	0.956 ± 0.03	−20.7
AMNP_GA1_GOx5/1	5_1	263 ± 6.89	0.293 ± 0.009	−18.8
AMNP_GA1_GOx10/1	10_1	790 ± 20.7	0.602 ± 0.01	−18.4
AMNP_GA1_GOx15/1	15_1	2929 ± 77.88	0.713 ± 0.02	−18.3
AMNP_SA0.5	-	168 ± 5.06	0.35 ± 0.01	−30.6
AMNP_SA0.5_GOx5/1	5_1	190 ± 5.2	0.321 ± 0.009	−25.6
AMNP_SA0.5_GOx10/1	10_1	181 ± 4.43	0.234 ± 0.007	−26.8
AMNP_SA0.5_GOx15/1	15_1	226 ± 8.78	0.375 ± 0.011	−29.9

**Table 3:** Parameters obtained from thermogravimetric analysis of simple and enzyme-coated nanoparticles.

Sample	Stage	T_onset_	T_peak_	T_endset_	W (%)	Residue (%)
MNP	I	36	70.6	93.1	12.3	80.8
GOx	I	32.5	69.5	129.5	6.9	33.8
	II	172.6	198.6	212	1.4	
	III	221	238.9	247	2.81	
	IV	256.9	297.7	672.5	51.9	
AMNP_SA0/5_GOx5/1	I	32.9	127.9	185.4	4.3	78.1
	II	190.4	287.9	565.4	13.3	

## Data Availability

The authors confirm that the data supporting the findings of this study are available within the article.
